# Optical Properties of Tensilely Strained Ge Nanomembranes

**DOI:** 10.3390/nano8060407

**Published:** 2018-06-06

**Authors:** Roberto Paiella, Max G. Lagally

**Affiliations:** 1 Department of Electrical and Computer Engineering and Photonics Center, Boston University, Boston, MA 02215, USA; 2 Department of Materials Science and Engineering, University of Wisconsin, Madison, WI 53706, USA; lagally@engr.wisc.edu

**Keywords:** nanomembranes, optical gain media, group-IV semiconductors, strain engineering

## Abstract

Group-IV semiconductors, which provide the leading materials platform of micro- electronics, are generally unsuitable for light emitting device applications because of their indirect- bandgap nature. This property currently limits the large-scale integration of electronic and photonic functionalities on Si chips. The introduction of tensile strain in Ge, which has the effect of lowering the direct conduction-band minimum relative to the indirect valleys, is a promising approach to address this challenge. Here we review recent work focused on the basic science and technology of mechanically stressed Ge nanomembranes, i.e., single-crystal sheets with thicknesses of a few tens of nanometers, which can sustain particularly large strain levels before the onset of plastic deformation. These nanomaterials have been employed to demonstrate large strain-enhanced photoluminescence, population inversion under optical pumping, and the formation of direct-bandgap Ge. Furthermore, Si-based photonic-crystal cavities have been developed that can be combined with these Ge nanomembranes without limiting their mechanical flexibility. These results highlight the potential of strained Ge as a CMOS-compatible laser material, and more in general the promise of nanomembrane strain engineering for novel device technologies.

## 1. Introduction

The development of group-IV semiconductor lasers that can be integrated within complex microelectronic systems with a CMOS-compatible process is a major goal of current research in optoelectronics. The key underlying challenge is the indirect-bandgap nature of the leading materials of Si-based microelectronics (i.e., Si, Ge, and their alloy SiGe), which results in extremely low light-emission efficiencies. The use of Ge as the laser active material is particularly compelling because of the relatively small difference (~0.14 eV) between its direct and fundamental-indirect bandgap energies. Ge is also more generally the subject of extensive renewed research interest [1]. Its large mobility of both electrons and holes is especially attractive in CMOS microelectronics, although challenges still exist related to the choice of gate dielectric and *n* doping [2]. In optoelectronics, near-infrared Ge photodetectors are already well-established [3].

A promising approach that is currently being pursued to overcome the indirect-bandgap nature of Ge for application to light emitting devices is based on strain engineering. Extensive theoretical work has shown that the introduction of tensile strain (larger than about 4% uniaxial or 1.9% biaxial for the standard (001) crystal orientation) has the effect of converting Ge into a direct-bandgap semiconductor capable of providing optical gain [4,5,6,7,8,9,10,11]. At the same time, the direct-bandgap energy of Ge under tensile strain is also red-shifted into the short-wave mid-infrared spectral region beyond 2 μm, where a wide range of biochemical sensing applications exists that could benefit strongly from an integrated lab-on-a-chip device platform. In passing, we note that the incorporation of Sn in Ge produces an effect similar to that of biaxial tensile strain (i.e., a decrease in the direct conduction-band minimum relative to the indirect valleys, and an overall red-shift in bandgap energy). While the growth of high-quality GeSn films remains quite challenging due to phase separation issues, recent progress has led to the demonstration of optically pumped lasing at cryogenic temperatures [12].

In semiconductor micro- and optoelectronics, strain is traditionally introduced by hetero- epitaxy, i.e., through the growth of sufficiently thin layers of the desired materials on a suitably lattice-mismatched template. In the case of Ge on Si, however, this approach is impractical given the exceedingly large mismatch of about 4.2% between the Si and Ge equilibrium lattice constants, which essentially precludes pseudomorphic growth (and would in any case produce compressively rather than tensilely strained Ge). While tensilely strained Ge can be grown on III-V semiconductor templates such as InGaAs [13,14], the main driver for the development of strained-Ge laser materials is their direct compatibility with Si substrates. As a result, novel straining techniques have been widely investigated in the past few years [15,16,17,18,19,20,21,22,23,24,25,26,27,28,29,30], involving mechanically stressed Ge nanostructures mostly derived from Ge-on-insulator (GOI) wafers—i.e., Si substrates coated with a buried oxide layer (BOX) underneath a thin Ge film.

In this context, the use of Ge nanomembranes (NMs) has proved to be particularly appealing as a means to studying the basic structural and optical properties of Ge under large biaxial tensile strain. Semiconductor NMs in general are single-crystal films with lateral dimensions in the μm- or mm-scale and thicknesses as small as several tens of nm, which are typically released from their native substrate via the selective etch of an underlying sacrificial layer. By virtue of their crystalline nature and unusual aspect ratios, these films can combine the exceptional electronic and optical properties of inorganic semiconductors with the extreme mechanical flexibility of soft polymeric materials. As a result, they are promising for a wide range of applications in flexible electronics and optoelectronics, as well as for fundamental studies in strain engineering [31].

This article reviews recent work on mechanically stressed Ge NMs aimed at investigating their basic optical properties and demonstrating the suitability of tensilely strained Ge as a Si-compatible laser gain medium [17,20,22,23,28]. The electronic band structure and calculated optical gain spectra of strained Ge are described in Section 2. In Section 3, we briefly review various straining techniques that have been applied to Ge nanostructures, with emphasis on the use of NMs to obtain wide-area biaxial tensile strain. Section 4 is focused on our experimental work based on the latter approach, including the demonstration of strain-enhanced photoluminescence, population inversion under optical pumping, and the formation of direct-bandgap Ge. Finally, in Section 5, we describe recent efforts aimed at the development of mechanically flexible optical cavities that can be integrated with NM active layers without limiting their mechanical flexibility.

## 2. Electronic and Optical Properties of Strained Ge

**Figure 1:**
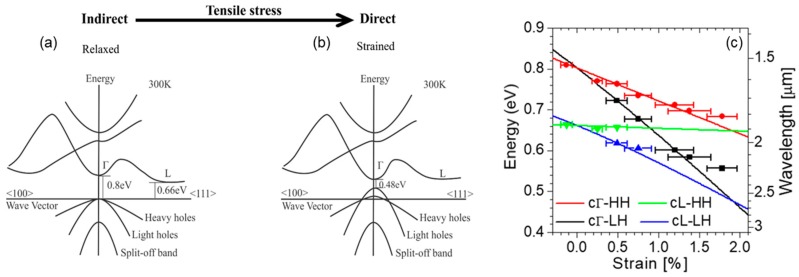
Strain-induced modifications of the Ge band structure. (**a**) Schematic band structure of unstrained Ge; (**b**) Schematic band structure of Ge under 1.9% biaxial tensile strain Reproduced with permission from [23]. American Chemical Society, 2014; (**c**) Solid lines: calculated bandgap energies between the Γ or L conduction-band minima and the HH or LH valence-band maxima of Ge as a function of biaxial strain. Symbols: peak emission energies extrapolated from the strain-resolved luminescence spectra shown in Figure 4a below. Reproduced with permission from [17]. National Academy of Sciences, 2011.

Unstrained Ge is an indirect-bandgap semiconductor, where the absolute minimum of the conduction band and the maxima of the valence bands occur, respectively, at the L and Γ points of reciprocal space (Figure 1a). At the same time, the conduction band also features a local minimum at Γ. The introduction of tensile strain in this material has the effect of lowering all conduction-band minima, but at different rates, with the Γ minimum decreasing more rapidly with increasing strain compared to the L valleys. When the strain exceeds about 1.9% (biaxial), the Γ and L minima cross over and Ge becomes a direct-bandgap semiconductor (Figure 1b). At the same time, the degeneracy of the valence bands at the Γ point is also lifted, with the light-hole (LH) band pushed up in energy relative to the heavy-hole (HH) one. The resulting variations in bandgap energies between all the conduction- and valence-band edges just discussed, as computed by deformation-potential theory [32], are plotted in Figure 1c. 

**Figure 2:**
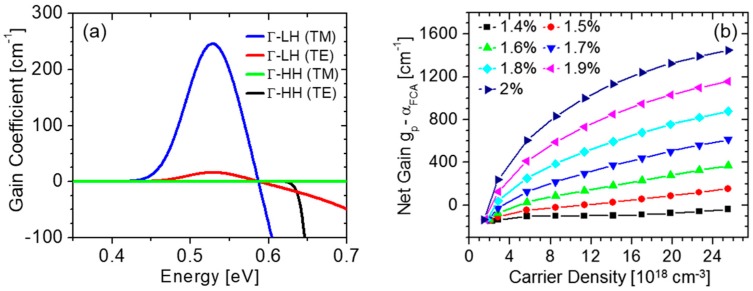
Optical gain properties of tensilely strained Ge. (**a**) Calculated TE and TM optical-gain spectra due to cΓ-LH and cΓ-HH transitions in Ge under 1.78-% biaxial tensile strain, in the presence of a density of injected carriers of 3.9 × 10^18^ cm^−3^. These values of strain and carrier density correspond to the experimental data shown in Figure 4b below. Reproduced with permission from [20]. John Wiley and Sons, 2012; (**b**) Peak gain coefficient of undoped Ge (including the effect of free-carrier absorption) plotted as a function of injected carrier density for different strain values. Reproduced with permission from [28]. AIP Publishing LLC, 2016.

When the direct conduction-band minimum lies below (or at least sufficiently close to) the L valleys, a population inversion at the Γ point can be established under practical pumping conditions. Extensive numerical simulations based on standard models of interband optical transitions in semiconductors [32] indicate that the resulting optical-gain values are adequate for laser operation [20]. Figure 2a shows representative gain spectra of strained Ge(001) due to conduction-to-LH (cΓ-LH) and conduction-to-HH (cΓ-HH) transitions, for both TE and TM polarized light (i.e., with electric field parallel and perpendicular to the plane of the biaxial strain, respectively). As shown in these plots, optical gain is only produced by the cΓ-LH transitions, which is a consequence of the aforementioned lifting of the valence-band degeneracy under tensile strain, which pushes the LH band above the HH one. As a result, in the presence of external carrier injection, the hole population in tensilely strained Ge mostly resides in the LH band. Due to the well-established polarization selection rules of interband transitions in semiconductors [32], the oscillator strength of cΓ-LH transitions is significantly larger for TM-polarized light, as also observed in Figure 2a. 

The peak gain coefficient g_p_ increases with both carrier density and tensile strain, as illustrated in Figure 2b [28]. To evaluate the full impact of increasing the carrier density *N* in tensilely strained Ge, this figure shows the net gain g_p_-α_FCA_, where the free-carrier absorption coefficient α_FCA_ also increases (linearly) with *N*. For the NM cavity geometry described in Section 5 below, the threshold gain required for lasing is estimated to be a few 100 cm^−1^ (if all relevant loss mechanisms are properly minimized). The simulation results of Figure 2b therefore indicate that such a threshold can be reached for strain values larger than about 1.6% biaxial. The larger the strain, the smaller the required density of injected carriers, as expected based on the corresponding decrease in direct versus indirect bandgap energy.

## 3. Ge-Nanostructure Straining Techniques

**Figure 3:**
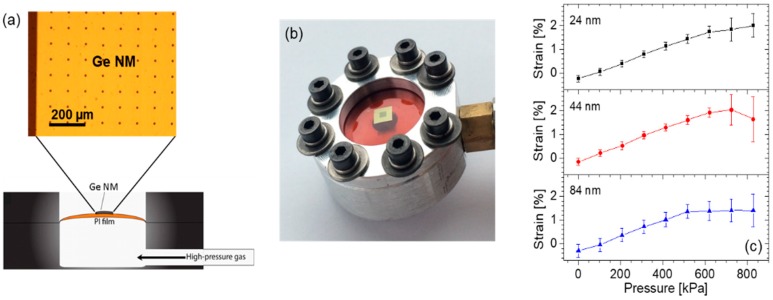
Mechanically stressed Ge NMs. (**a**) Schematic illustration of the experimental setup used to introduce biaxial tensile strain in Ge NMs. A top-view optical micrograph of a NM bonded onto a PI film is also shown. The periodic black dots are etchant access holes, used to facilitate the Ge NM release from its GOI substrate. Reproduced with permission from [23]. American Chemical Society, 2014; (**b**) Photograph of the sample mount under an applied gas pressure of 520 kPa. The Ge NM (here containing an overlaying pillar array, as described in Section 5 below) is visible near the center of the PI film. Reproduced with permission from [28]. AIP Publishing LLC, 2016; (**c**) Strain/stress curves measured by Raman spectroscopy on Ge NMs with three different thicknesses. Reproduced with permission from [17]. National Academy of Sciences, 2011.

The Ge NMs used in the work reviewed below are fabricated by releasing the Ge template layer of a commercial GOI wafer [33]. Specifically, the membrane boundaries and etchant access holes are first patterned in this Ge layer using photolithography and reactive ion etching. Next, the underlying BOX layer is completely removed with a wet etch in a hydrofluoric-acid solution. The released Ge NMs are then bonded onto flexible films of polyimide (PI) using a wet transfer procedure. For the strain-dependent measurements described below, the PI films with the attached NMs are integrated in a metallic chamber which is then pressurized with a controlled gas inflow. In this configuration, the NM effectively sits on the surface of an expanding sphere, so that the resulting strain is fully biaxial. A schematic illustration of the experimental setup (with an optical micrograph of a NM bonded onto a PI film) and a photograph of the pressure cell are shown in Figure 3a,b, respectively.

The key property of NMs that allows them to reach particularly large strain levels before the onset of plastic deformation is provided by their nanoscale thicknesses. This idea is confirmed and quantified by the Raman measurement results of Figure 3c [17], where the strain introduced in the Ge lattice with the setup of Figure 3a,b is measured as a function of applied gas pressure for three different NM thicknesses. In all three samples, the strain initially increases linearly with applied stress, and then begins to saturate because of the formation of cracks that allow for local strain relaxation in their immediate vicinity. Importantly, the thinner the NM, the larger the strain threshold for the formation of such cracks. In particular, for the smallest NM thickness considered in these measurements (24 nm), a nearly linear strain/stress relation is observed up to a maximum strain value of over 2% (averaged over several random sites on the NM), where Ge is expected to feature a direct fundamental bandgap.

The straining procedure just described is particularly convenient for the purpose of material characterization studies, as it produces large, uniform, and dynamically tunable strain across macroscopic sample areas. Therefore, it provides an ideal platform to study the optical properties of strained Ge reviewed in this article. For practical applications, similar results can in principle be obtained with integrated NM devices on Si chips using MEMS technology [8], piezoelectrics [34], or microfluidics [35,36].

A wide range of other straining techniques has also been investigated. In some of the earliest work in this area, tensile strain has been introduced in plastically relaxed Ge films grown on Si by taking advantage of the different thermal expansion coefficients of Si and Ge with an annealing process [37]. While the resulting strain is quite small (less than about 0.3%), this approach has been used in conjunction with degenerate *n*-type doping (to populate the Γ valley) to demonstrate electrically pumped Ge lasers [38]. To date, however, the performance of these devices remains quite limited, likely due to the excessive free-carrier absorption and Auger recombination losses caused by the degenerate doping. 

More recently, significant research efforts have also focused on the use of stressor layers with different types of Ge nanostructures [18,19,21,24,25,26,27,29,30]. Typically, these layers consist of material deposited under large compressive strain, which is then allowed to relax partially via elastic strain sharing with an attached partially suspended Ge film. As a result, tensile strain is introduced in the Ge film. Specific stressor materials that have been employed for this purpose include Si_3_N_4_ [18,25,27,29] and tungsten [19], deposited on a suitably patterned Ge film. Another approach is based on the small (~0.2–0.3%) tensile strain that exists in Ge films grown on Si (after the aforementioned annealing process) or in the Ge template layers of typical GOI substrates. If constricted structures (e.g., in the shape of suspended microbridges) are then patterned in this Ge layer, their local strain can be significantly amplified compared to the surrounding regions, because stress is inversely proportional to cross-sectional area. With this approach, uniaxial and biaxial strain values up to 3.1% [21] and 1.9% [26], respectively, have been measured in micron-scale regions.

These techniques based on built-in stress are directly compatible with on-chip integration, and device applications are already being explored. In [27], high Q-factor Ge microdisk cavities (combining a Si_3_N_4_ stressor layer with circular Bragg reflectors) have been developed, leading to the observation of line narrowing under optical pumping. A similar microdisk geometry (but without the Bragg reflectors) has also been reported with GeSn [29] as a way to combine the beneficial effects of both tensile strain and Sn incorporation. Finally, an optically pumped laser operating at cryogenic temperatures has also been demonstrated recently [30], based on the suspended-microbridge geometry just described combined with a pair of distributed Bragg mirrors.

## 4. Strained-Enhanced Light Emission from Mechanically Stressed Ge Nanomembranes

**Figure 4:**
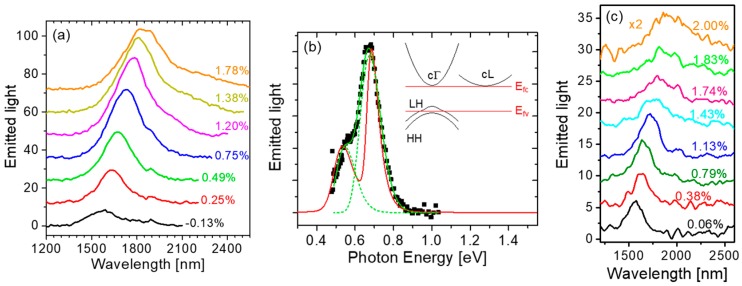
Strain-enhanced light emission from Ge NMs. (**a**) Room-temperature PL spectra of a 40-nm-thick undoped Ge NM at different levels of biaxial tensile strain; (**b**) Symbols: PL spectrum of the NM of (a) (normalized to the spectral response of the measurement setup) at a strain of 1.78%. Green lines: Gaussian fits showing the cΓ-HH and cΓ-LH contributions (dashed) and their sum (solid). Red line: calculated spontaneous emission spectrum. Inset: schematic band diagram of Ge at a strain of 1.78%, and estimated positions of the quasi-Fermi levels relative to the band edges in the presence of the PL pump pulses; (**c**) Room-temperature PL spectra of a 24-nm-thick undoped Ge NM at different levels of biaxial tensile strain. The vertical axes in (**a**,**c**) are in arbitrary units, and the different spectra are shifted vertically relative to one another for the sake of illustration clarity. The PL pump light in these measurements was provided by an optical parametric oscillator with 5-ns pulse width, 20-Hz repetition rate, 960-nm wavelength, and 3-mW average power. Reproduced with permission from [17]. National Academy of Sciences, 2011.

The light-emission properties of mechanically stressed Ge(001) NMs are illustrated by the photoluminescence (PL) data shown in Figure 4 [17,20]. As the applied biaxial tensile strain is increased, the NM emission spectrum is strongly enhanced and simultaneously red-shifted (Figure 4a). This behavior is of course consistent with the corresponding decrease in the Ge direct-bandgap energy relative to the indirect one, which causes a larger and larger fraction of the injected electrons to relax near the Γ minimum of the conduction band. The measured luminescence spectra can be fitted with multiple Gaussian peaks, corresponding to electronic transitions from the direct and indirect minima of the conduction band to the maxima of the strain-split LH and HH valence bands. The emission photon energies extrapolated with this procedure are in good agreement with the calculated bandgap energies of the same transitions, as illustrated in Figure 1c, where the symbols were obtained from the spectra of Figure 4a.

The same data analysis also indicates that the measured emission spectra of mechanically stressed Ge NMs are dominated by transitions involving HHs, even though under tensile strain the LH band resides at higher energy, and therefore has a higher hole population, compared to the HH band (Figure 1b). This behavior is a result of the aforementioned polarization selection rules: electronic transitions into the LH valence band mostly generate TM-polarized photons that propagate on the plane of the NM and thus cannot be detected in standard surface-emission PL measurements. For the same reason, the full increase in radiative efficiency produced by the applied strain cannot be fully quantified based on these measurement results.

The luminescence spectra measured at high strain also provide evidence of population inversion in the Ge NMs. The key observation in this respect is the ability to resolve in the high-strain emission spectra two separate peaks due to transitions into both LH and HH valence bands near the Γ point (Figure 4b). The relative height of these two peaks depends uniquely on the density of holes injected into the NM under optical pumping. This parameter can therefore be extrapolated by fitting the emission spectra with a standard model of light emission in (strained) semiconductors [32], and then compared to the transparency carrier density of the same NM evaluated with the same model. With this analysis, we find that when the applied strain is above ~1.4% [20], a population inversion is established in the NMs under study.

Finally, Figure 4c shows the strain-resolved PL spectra of the thinnest (24 nm) NM measured in this work. The luminescence intensity of this sample is relatively weak, because of the reduced pump-light absorption and increased nonradiative surface recombination in ultrathin NMs. At the same time, however, the applied strain can be increased to over 2% (where Ge is expected to become a direct-bandgap semiconductor), before the onset of extended-defect formation indicated by the saturation in the strain/stress curve. The emission spectrum measured with this sample at 2% biaxial strain is consistent with the calculated cΓ-HH transition energy in the direct-bandgap regime. Therefore, the results of Figure 4c in conjunction with the Raman data of Figure 3c indicate the formation of direct-bandgap Ge in this NM.

## 5. Flexible Nanomembrane Optical Cavities

**Figure 5:**
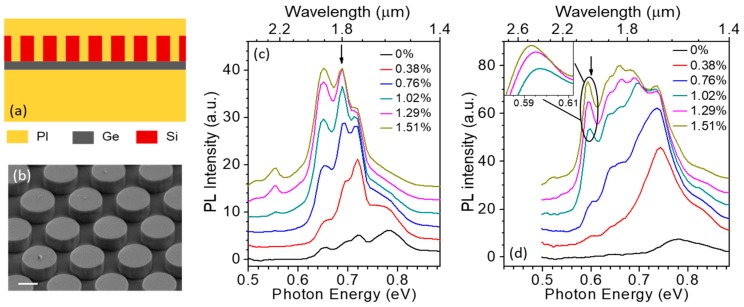
Flexible photonic-crystal cavities for tensilely strained Ge NMs. (**a**) Schematic device cross section; (**b**) SEM image of a Si-pillar array before transfer onto a Ge NM on PI. The scale bar is 500 nm; (**c**,**d**) Strain-resolved room-temperature PL spectra measured on two Ge(001) samples with different column periods *a* and diameters D: (**c**) *a* = 1060 nm, D = 850 nm; (**d**) *a* = 1340 nm, D = 950 nm. Inset of (**d**): zoom-in of the features within the black ellipse, showing strain tuning of the cavity modes. The arrow in each plot indicates the calculated photon energy of the main TM-polarized cavity resonance. (**b**–**d**): Reproduced with permission from [28]. AIP Publishing LLC, 2016.

The theoretical and experimental results reviewed in the previous sections substantiate the suitability of mechanically stressed Ge NMs for laser applications. An important challenge in the development of these devices is related to the nanoscale thickness of the NMs, which is too small to provide by itself the required in-plane waveguiding of the (TM-polarized) emitted light. As a result, additional guiding layers must be added to the NMs, without at the same time compromising the NM mechanical flexibility (which is essential to enable gain through straining). This challenge has been addressed with the device geometry shown schematically in Figure 5a, where a periodic array of dielectric columns is deposited on the NM on PI [22,28]. These arrays can be made thick enough and with sufficiently large average refractive index to act as the core guiding layers. At the same time, if their periodicity matches the emission wavelength, they can also provide vertical outcoupling and in-plane optical feedback of the emitted light, by first and second-order diffraction, respectively. Finally, by virtue of their disconnected geometry, the same arrays will not limit the maximum strain that can be introduced in the underlying NM (at least in the regions between neighboring pillars).

In recent work, the device geometry just described has been implemented with a novel fabrication process based on direct NM assembly [28]. In this process, an array of Si columns embedded in a PI film is patterned in the template layer of a commercial Si-on-insulator (SOI) wafer, released from its native substrate with a selective wet etch of the SOI BOX layer, and then transferred onto a previously prepared Ge NM on PI. The resulting pillars have extremely smooth sidewalls (Figure 5b) and therefore can be expected to provide minimal optical-scattering losses. Furthermore, because they are based on single-crystal Si, they also feature negligible absorption losses at the strained-Ge emission wavelength.

A large increase and red shift in PL emission with increasing strain is once again observed with these devices, indicating a similar degree of mechanical flexibility as in the bare Ge NMs described above. Representative results are shown in Figure 5c,d for two 50-nm-thick Ge NMs coated with arrays of different periods. In fact, a significantly larger enhancement in PL efficiency is obtained with these devices (up to 12× in Figure 5d) compared to bare NMs, due to the efficient vertical outcoupling of the TM-polarized emission produced by the column array. Furthermore, a complex pattern of relatively narrow features is observed in the PL spectra of Figure 5, associated with different modes of the photonic-crystal cavity provided by the pillars. The spectral positions of these features are in good agreement with numerical simulations of the photonic-crystal band structures [28], and can be tuned by varying the array period and the applied strain (inset of Figure 5d). Altogether, these results therefore demonstrate that ultrathin NM active layers can be effectively coupled to an optical cavity while still fully preserving their mechanical flexibility.

## 6. Conclusions

This article has reviewed recent work, spanning approximately eight years, on mechanically stressed Ge NMs, aimed at the investigation of their ability to sustain large amounts of tensile strain and correspondingly provide enhanced light emission efficiency and optical gain for laser applications. Because of their extreme aspect ratios, these films can be strained beyond the threshold for the formation of direct-bandgap Ge. Numerical simulations indicate that the resulting optical-gain properties are comparable to those of traditional III-V semiconductor laser materials. The expected increase in luminescence efficiency with increasing strain has been measured with NMs of different thicknesses, together with a large red shift of the emission spectra towards the short-wave mid-infrared region. The same experimental results also reveal the presence of population inversion under optical pumping in NMs strained above ~1.4%. Finally, photonic-crystal cavities fully compatible with the flexibility requirements of these NM active layers have been developed, based on a novel membrane-assembly process. 

These results in general highlight the potential of strained Ge as a CMOS-compatible laser material. Furthermore, they are promising for the future development of mechanically stressed Ge NM lasers, which could then be integrated on Si chips using MEMS or related technologies. The key remaining challenge is likely related to minimizing the optical losses in the cavity devices of Figure 5, which have not yet shown evidence of spectral narrowing of the emission peaks with increasing pumping and/or strain. In particular, an important loss mechanism appears to be scattering by defects in the NM originating from the GOI template layer, whose structural quality is therefore of critical importance for future progress in this research. In fact, such defects also tend to act as crack initiation sites under mechanical stress, so that their minimization would also allow increasing the maximum achievable strain.
